# What is the Best Radionuclide for Immuno-PET of Multiple Myeloma? A Comparison Study Between ^89^Zr- and ^64^Cu-Labeled Anti-CD138 in a Preclinical Syngeneic Model

**DOI:** 10.3390/ijms20102564

**Published:** 2019-05-24

**Authors:** Clément Bailly, Sébastien Gouard, François Guérard, Benjamin Chalopin, Thomas Carlier, Alain Faivre-Chauvet, Patricia Remaud-Le Saëc, Mickaël Bourgeois, Nicolas Chouin, Latifa Rbah-Vidal, Raphaël Tripier, Ferid Haddad, Françoise Kraeber-Bodéré, Caroline Bodet-Milin, Michel Chérel

**Affiliations:** 1 CRCINA, INSERM, CNRS, Université d’Angers, Université de Nantes, 44093 Nantes, France; clement.bailly@chu-nantes.fr (C.B.); sebastien.gouard@univ-nantes.fr (S.G.); francois.guerard@univ-nantes.fr (F.G.); benjamin.chalopin@yahoo.fr (B.C.); thomas.carlier@chu-nantes.fr (T.C.); alain.faivre-chauvet@univ-nantes.fr (A.F.-C.); patricia.lesaec@univ-nantes.fr (P.R.-L.S.); mickael.bourgeois@nantes.inserm.fr (M.B.); nicolas.chouin@oniris-nantes.fr (N.C.); latifa.rbah-vidal@univ-nantes.fr (L.R.-V.); francoise.bodere@chu-nantes.fr (F.K.-B.); caroline.milin@chu-nantes.fr (C.B.-M.); 2 Nuclear Medicine Department, University Hospital, 44093 Nantes, France; 3 Groupement d’Intérêt Public Arronax, 44800 Saint-Herblain, France; ferid.haddad@univ-nantes.fr; 4 Oniris, Ecole Nationale Vétérinaire, Agroalimentaire et de l’alimentation de Nantes-Atlantique, 44300 Nantes, France; 5 CNRS-UMR6521, University of Bretagne Occidentale, 29238 Brest, France; raphael.tripier@univ-brest.fr; 6 Nuclear Medicine Department, ICO-René Gauducheau Cancer Center, 44800 Saint-Herblain, France

**Keywords:** multiple myeloma, immuno-PET, copper-64, zirconium-89, murine CD138

## Abstract

Although positron emission tomography (PET) imaging with 18-Fluorodeoxyglucose (^18^F-FDG) is a promising technique in multiple myeloma (MM), the development of other radiopharmaceuticals seems relevant. CD138 is currently used as a standard marker for the identification of myeloma cells and could be used in phenotype tumor imaging. In this study, we used an anti-CD138 murine antibody (9E7.4) radiolabeled with copper-64 (^64^Cu) or zirconium-89 (^89^Zr) and compared them in a syngeneic mouse model to select the optimal tracers for MM PET imaging. Then, 9E7.4 was conjugated to TE2A-benzyl isothiocyanate (TE2A) and desferrioxamine (DFO) chelators for ^64^Cu and ^89^Zr labeling, respectively. ^64^Cu-TE2A-9E7.4 and ^89^Zr-DFO-9E7.4 antibodies were evaluated by PET imaging and biodistribution studies in C57BL/KaLwRij mice bearing either 5T33-MM subcutaneous tumors or bone lesions and were compared to ^18^F-FDG-PET imaging. In biodistribution and PET studies, ^64^Cu-TE2A-9E7.4 and ^89^Zr-DFO-9E7.4 displayed comparable good tumor uptake of subcutaneous tumors. On the bone lesions, PET imaging with ^64^Cu-TE2A-9E7.4 and ^89^Zr-DFO-9E7.4 showed higher uptake than with ^18^F-FDG-PET. Comparison of both 9E7.4 conjugates revealed higher nonspecific bone uptakes of ^89^Zr-DFO-9E7.4 than ^64^Cu-TE2A-9E7.4. Because of free ^89^Zr’s tropism for bone when using ^89^Zr-anti-CD138, ^64^Cu-anti-CD138 antibody had the most optimal tumor-to-nontarget tissue ratios for translation into humans as a specific new imaging radiopharmaceutical agent in MM.

## 1. Introduction

Over the last 30 years, major advances have been made with regard to the management of multiple myeloma (MM) [1,2]. These improvements have occurred along with our evolving understanding of this malignancy [3]. Multi-clonal heterogeneity of MM still remains one of the main challenges in developing effective therapeutic strategies [4]. Immunologic approaches represent an attractive solution to address this issue for treatment [5], however also for imaging [6] in the context of theranostic approaches. Indeed, the combination of positron emission tomography (PET) with monoclonal antibodies (mAbs) enables the realisation of a specific imaging called immuno-PET [7]. Among the interesting targets, CD138 or syndecan-1 is a cell surface proteoglycan that plays a critical role in the interaction between MM cells and their microenvironment [8,9]. This antigen is currently used as a standard marker for the identification and purification of MM cells in daily practice. Anti-CD138 immuno-PET thus has the potential to improve MM imaging, especially regarding lesions with low metabolic activity [10,11]. Moreover, it could also be considered as a companion agent for currently developed therapies targeting CD138 [12,13]. In the past several years, our group has also proven that radioimmunotherapy (RIT) combining anti-CD138 mAb and alpha-emitters radionuclides is effective in an immuno-competent preclinical MM model and is feasible in humans [14,15].

For immuno-PET, the choice of the radionuclides remains a fundamental question [7]. Combining appropriate half-lives for mAbs biodistribution and favorable emission properties for imaging, Copper-64 (^64^Cu) and Zirconium-89 (^89^Zr) have monopolized much of the researchers’ attention during the last decade with an advantage for the second one in terms of number of studies [16,17]. However, reported release of ^89^Zr from the imaging probe may represent a drawback for bone lesions imaging [18] and therefore for MM assessment. In this work, we report the preclinical evaluation of a novel PET imaging agent based on the ^89^Zr-labeled anti-mouse syndecan-1 mAb (9E7.4, IgG2a κ isotype) [19] in a subcutaneous model and a bone marrow disseminated MM model using desferrioxamine B (DFO) as chelator. This is compared to ^18^F-FDG-PET and bioluminescence imaging as gold standards and to ^89^Zr-oxalate imaging as a control of potential ^89^Zr release by the chelator agent. Furthermore, given our previous experience using ^64^Cu [11] and to establish the optimal radiolabeled 9E7.4 mAb for immuno-PET, biodistribution and PET imaging in vivo of ^89^Zr- and ^64^Cu-mAb conjugates have been compared with emphasis on bone uptake.

## 2. Results

To evaluate and select the optimal radiolabeled 9E7.4 mAb for immuno-PET of MM lesions in bones, we have generated two radio-immunoconjugates PET tracers (^89^Zr-DFO-9E7.4 and ^64^Cu-TE2A-9E7.4). Biodistribution and imaging studies were performed. This report focuses on PET imaging with ^89^Zr-DFO-9E7.4 and follows a recent published work on ^64^Cu-TE2A-9E7.4 [11]. However, comparison between both in a disseminated model is presented.

### 2.1. Ex Vivo Biodistribution Experiments

**Figure 1:**
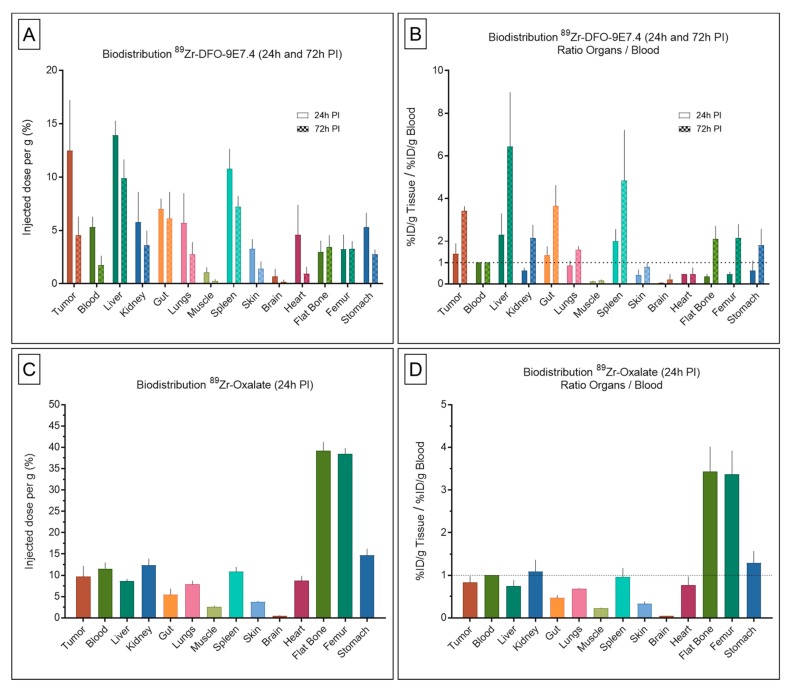
Biodistribution results and organ-to-blood ratios of ^89^Zr-DFO-9E7.4, ^89^Zr-oxalate in tumor-bearing mice. Ex vivo biodistribution results (**A**) and organ-to-blood ratios (**B**) of ^89^Zr-DFO-9E7.4 at 24 h and 72 h post-injection (PI), in the subcutaneous tumor model (*n* = 3 for each group). Ex vivo biodistribution results (**C**) and organ-to-blood ratios (**D**) of ^89^Zr-oxalate at 24 h PI (*n* = 3). Values are expressed in percentage of the injected radioactive dose per gram of tissue (%ID/g) and presented as mean ± SD.

Ex vivo biodistribution at 24 h and 72 h post-injection (PI) results are presented in Figure 1 and Table 1. On the study conducted 24 h after administration of ^89^Zr-DFO-9E7.4 (Figure 1A,B) in a subcutaneous model of MM, the tracer displayed correct accumulation in the tumors which decreased at 72 h PI. ^89^Zr-DFO-9E7.4 showed significant blood clearance from 24 h PI to 72 h PI, resulting in increased tumor-to-blood ratios. The radio-immunoconjugate also showed relative high uptakes of ^89^Zr-DFO-9E7.4 in several normal organs such as liver, spleen and guts. Low muscle uptake was found at both 24 h and 72 h PI. All other organs displayed activity concentrations of 5 %ID/g or less at 24 h with decreasing activity at 72 h PI. Only flat bones and femurs showed increasing activity between 24 h and 72 h PI. As a control of potential ^89^Zr release, biodistribution experiments at 24 h PI of ^89^Zr-oxalate were performed (Figure 1C,D). These showed high activity in the bones and relative high uptakes in several normal organs including blood and tumors resulting in very poor tumor-to-blood ratios.

### 2.2. Small Animal PET and CT Imaging

#### 2.2.1. PET Imaging of Subcutaneous Tumor

**Figure 2:**
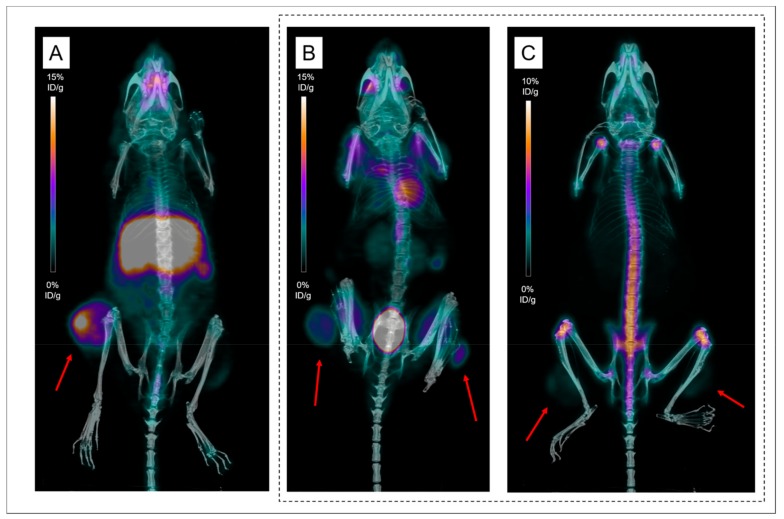
Positron emission tomography (PET) imaging with ^89^Zr-DFO-9E7.4, ^18^F-FDG-PET and ^89^Zr-oxalate in tumor-bearing mice. Maximum intensity projections of PET and CT imaging at 24 h post-injection (PI) (**A**) of Mouse 1 showing uptake of a subcutaneous tumor (Tumors are indicated by red arrows). Maximum intensity projections of PET and CT imaging with ^89^Zr-oxalate at 24 h PI (**C**) of Mouse 10 showing low uptakes in both subcutaneous tumors while clearly visible on maximum intensity projections of ^18^F-FDG-PET and CT imaging (**B**).

**Figure 3:**
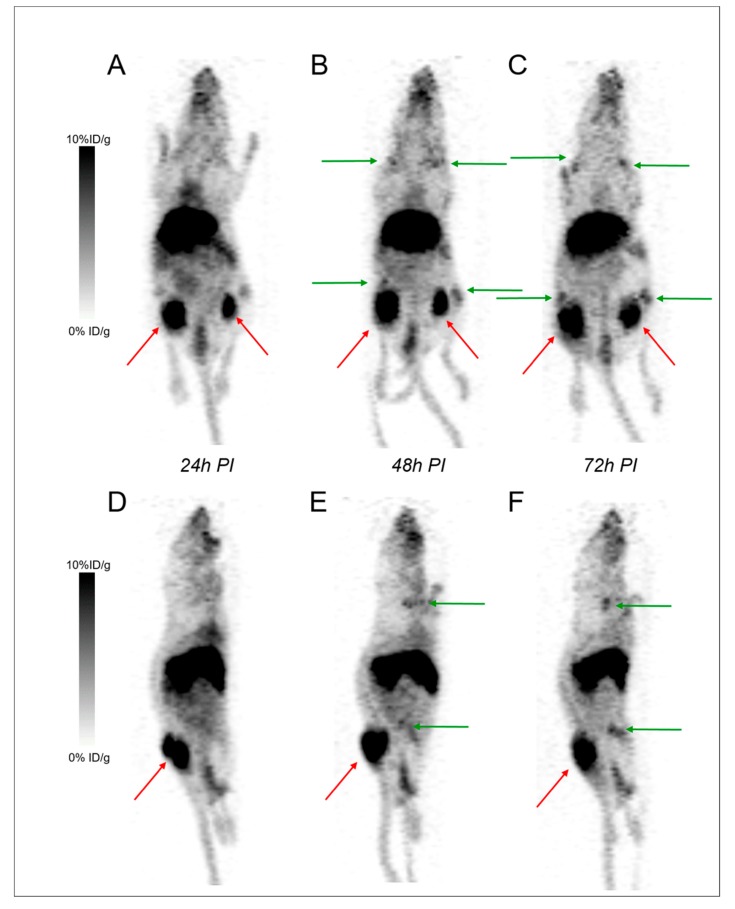
PET imaging with ^89^Zr-DFO-9E7.4 in the subcutaneous (SC) model. Maximum intensity projections of PET imaging at 24 h post-injection (PI) (**A**;**D**), 48 h PI (**B**;**E**) and 72 h PI (**C**;**F**) showing uptake of a subcutaneous tumor and of an inguinal lymph node (Tumors are indicated by red arrows) (Mouse 8). Green arrows showed conspicuous binding of free ^89^Zr over time at sites of osseous turnover.

In vivo, targeting of MM lesions by ^89^Zr-DFO-9E7.4 was firstly evaluated in a syngeneic model of mice bearing subcutaneous tumors. PET imaging experiments (Figure 2 and Figure 3) confirmed biodistribution observations and helped to visualize in vivo distributions of ^89^Zr-DFO-9E7.4 over time. On PET images obtained at 24 h PI, subcutaneous (SC) tumors were clearly visible with ^89^Zr-DFO-9E7.4 (Figure 2A) while presenting very modest accumulation with ^89^Zr-oxalate (Figure 2C), with ^18^F-FDG-PET being used as control imaging (Figure 2B). Figure 3 illustrates the progressive decreasing contrast of SC tumors at 24 h, 48 h and 72 h PI and the conspicuous increasing bones uptake (predominant on coxo-femoral joints, shoulders and knees). High liver, spleen and intestines uptakes were observed at all the time points. Because of this ^89^Zr accumulation in bones, as early as 48 h PI, 24 h PI time-point seems the optimal one for immuno-PET imaging with this radio-immunoconjugate.

#### 2.2.2. PET Imaging of Disseminated Disease

To establish a model of disseminated disease, mice were injected intravenously via a tail vein and the distribution was assessed using bioluminescence. Mice injected IV developed lesions in the skull, spine, sacrum, iliac wings and members (Table S1).

**Figure 4:**
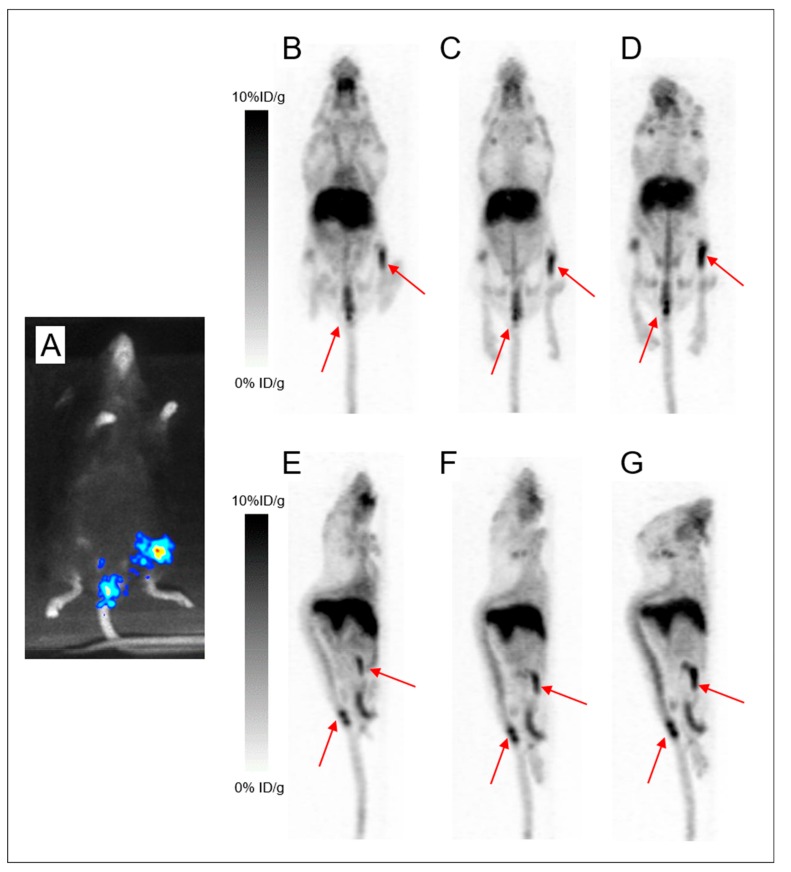
PET imaging with ^89^Zr-DFO-9E7.4 in the disseminated model. Bioluminescence imaging (**A**) revealing lesions in the sacrum and the left femur. Maximum intensity projections of PET and CT imaging with ^89^Zr-DFO-9E7.4 at 24 h post-injection (PI) (**B**;**E**), 48 h PI (**C**;**F**), and 72 h PI (**D**;**G**) showing uptakes in the sacrum and left femur (Tumors are indicated by arrows) (Mouse 13).

PET imaging with ^89^Zr-DFO-9E7.4 was performed 24 h, 48 h and 72 h PI (Figure 4). Besides the physiological uptakes also observed in the SC model, bone metastases were easily distinguished with excellent tumor-to-background ratios. Table S1 shows the lesion territories for each imaging method. Imaging with ^89^Zr-DFO-9E7.4 was able to detect all the lesions observed with bioluminescence imaging. High contrast was observed at all time-points yet, as observed in the SC model, PET images that were realized at 24 h PI seemed less hampered by aspecific bone uptakes (Figure 4).

#### 2.2.3. Focus on Bones and Bone Marrows

**Figure 5:**
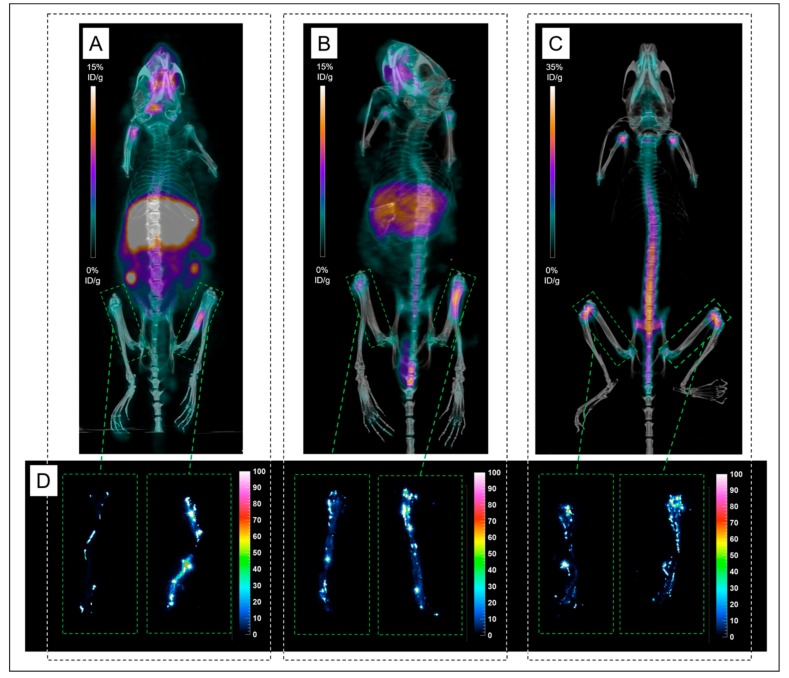
PET imaging and Digital autoradiography acquisitions performed on femurs of Mice imaged with ^89^Zr-DFO-9E7.4 or ^89^Zr-oxalate. Maximum intensity projections of PET and CT imaging with ^89^Zr-DFO-9E7.4 at 24 h post-injection (PI) (**A**) of Mouse 14 showing uptake in the left femur. Maximum intensity projections of PET and CT imaging with ^89^Zr-DFO-9E7.4 at 48 h PI (**B**) of Mouse 15 showing uptake in the left femur. Maximum intensity projections of PET and CT imaging with ^89^Zr-oxalate at 24 h PI (**C**) of Mouse 19. Digital autoradiography acquisitions performed on femurs of Mice 14, 15 and 19 (**D**).

A representative group of mice with femur lesions imaged at 24 h and 48 h PI were studied in more detail regarding bone uptake of ^89^Zr (Figure 5). Both femurs of the mice were harvested and digital autoradiography acquisitions were performed. An accumulation of activity was observed on PET and autoradiography images in mineralized constituents of the bones (compact bone and epiphysis) with similar uptakes between ^89^Zr-DFO-9E7.4 at 48 h PI and ^89^Zr-oxalate. This apparent strong affinity of ^89^Zr for the bones and joints resulted in a hampered contrast for visualization of the MM lesions in the bone marrow cellular compartment observed at 48 h PI compared to the images realized at 24 h PI (Figure 5).

**Figure 6:**
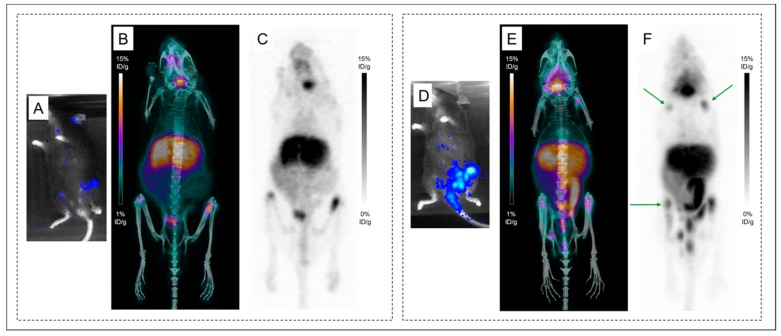
Altered contrast in the disseminated model imaged with ^89^Zr-DFO-9E7.4 at 24 h post-injection. Bioluminescence imaging (**A**) revealing lesions in both femurs. Maximum intensity projections of PET and CT imaging with ^89^Zr-DFO-9E7.4 at 24 h post-injection (PI) (**B**) and maximum intensity projections of PET imaging with ^89^Zr-DFO-9E7.4 at 24 h PI (**C**) showing uptake in both femurs (Mouse 16). Bioluminescence imaging (**D**) revealing lesions in the sacrum, left femur and both iliac wings. Maximum intensity projections of PET and CT imaging with ^89^Zr-DFO-9E7.4 at 24 h PI (**E**) and maximum intensity projections of PET imaging with ^89^Zr-DFO-9E7.4 at 24 h PI (**F**) showing uptake in the sacrum, left femur and both iliac wings (Mouse 17). Multiple false-positive osseous foci were also observed (indicated by green arrows).

Figure 6 illustrates the altered contrast observed in the disseminated model starting as early as 24 h PI. PET imaging with ^89^Zr-DFO-9E7.4 at 24 h PI of Mice 16 and 17 (Figure 6.B,C,E,F) showed multiple osseous uptakes corresponding to MM lesions as assessed by bioluminescence imaging, used as the gold standard. Yet symmetric joints’ uptake (shoulders and knees) was also observed on PET imaging of Mouse 17, corresponding to false-positive osseous foci due to free ^89^Zr.

### 2.3. Comparison of Both Radioimmunoconjugates

#### 2.3.1. In the Same Mouse

**Figure 7:**
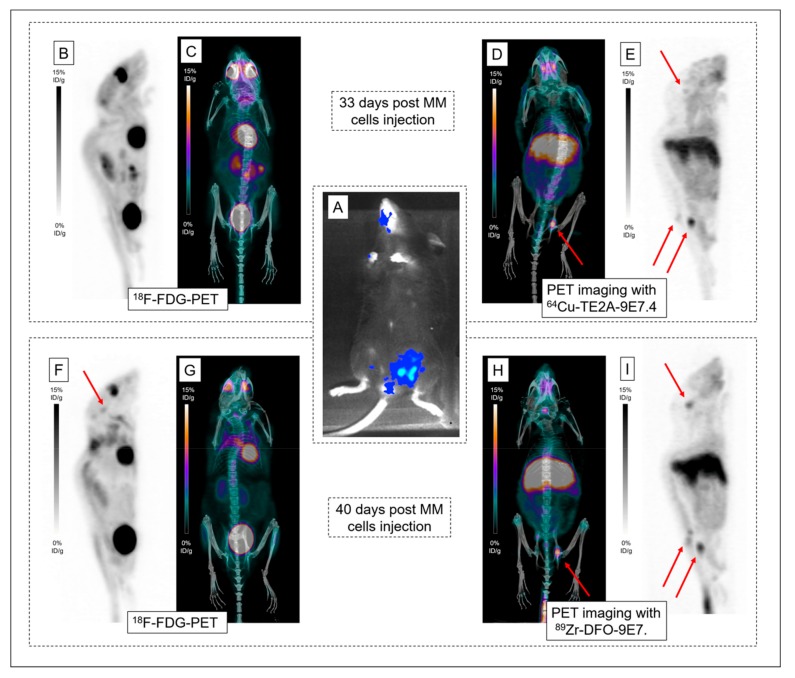
PET imaging with ^64^Cu-TE2A-9E7.4 and ^89^Zr-DFO-9E7.4. Bioluminescence imaging (**A**) revealing lesions in the spine and the left iliac wing. Maximum intensity projections of ^18^F-FDG-PET and CT (**B**;**C**) and maximum intensity projections of PET and CT imaging with ^64^Cu-TE2A-9E7.4 at 24 h post-injection (PI) (**D**;**E**), realized 33 days post multiple myeloma (MM) cells injection in Mouse 18. Maximum intensity projections of ^18^F-FDG-PET and CT (**F**;**G**) and maximum intensity projections of PET and CT imaging with ^89^Zr-DFO-9E7.4 at 24 h (PI) (**H**;**I**), realized 40 days post MM cells injection. Pathological uptakes were spotted by red arrows in the skull, spine and the left iliac wing.

To determine the best radio-immunoconjugate for CD138 immuno-PET imaging in MM bone lesions, ^89^Zr-labeled and ^64^Cu-labeled 9E7.4 were compared. ^64^Cu-TE2A-9E7.4 first and ^89^Zr-DFO-9E7.4 seven days after were used for serial imaging (Table S1) and high-contrast images showing specific tumor uptakes were obtained (Figure 7). PET images were collected at 24 h PI as optimal contrast was observed at this time point for both tracers. Both tracers were able to detect all the lesions observed with bioluminescence imaging. ^64^Cu-TE2A-9E7.4 was also able to detect skull infringement of the Mouse 18 (Figure 7E), undistinguishable on ^18^F-FDG-PET images (Figure 7B). Higher tumor-to-normal tissue contrast was observed on ^89^Zr-DFO-9E7.4, probably due to the seven-day interval between both immuno-PET imaging and the continuous tumor progression, as assessed by the skull infringement’s visualization on the second ^18^F-FDG-PET images (Figure 7F).

#### 2.3.2. Between Both Experiments

**Figure 8:**
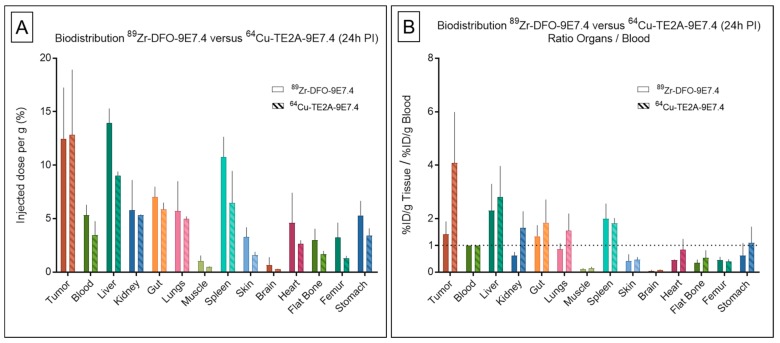
Biodistribution results and organ-to-blood ratios of ^89^Zr-DFO-9E7.4 and ^64^Cu-TE2A-9E7.4 in tumor-bearing mice. Ex vivo biodistribution results (**A**) and organ-to-blood ratios (**B**) of ^64^Cu-TE2A-9E7.4 and ^89^Zr-DFO-9E7.4 at 24 h post-injection (PI) in the subcutaneous tumor model (*n* = 3 for each group). Values are expressed in percentage of the injected radioactive dose per gram of tissue (%ID/g) and presented as mean ± SD.

**Figure 9:**
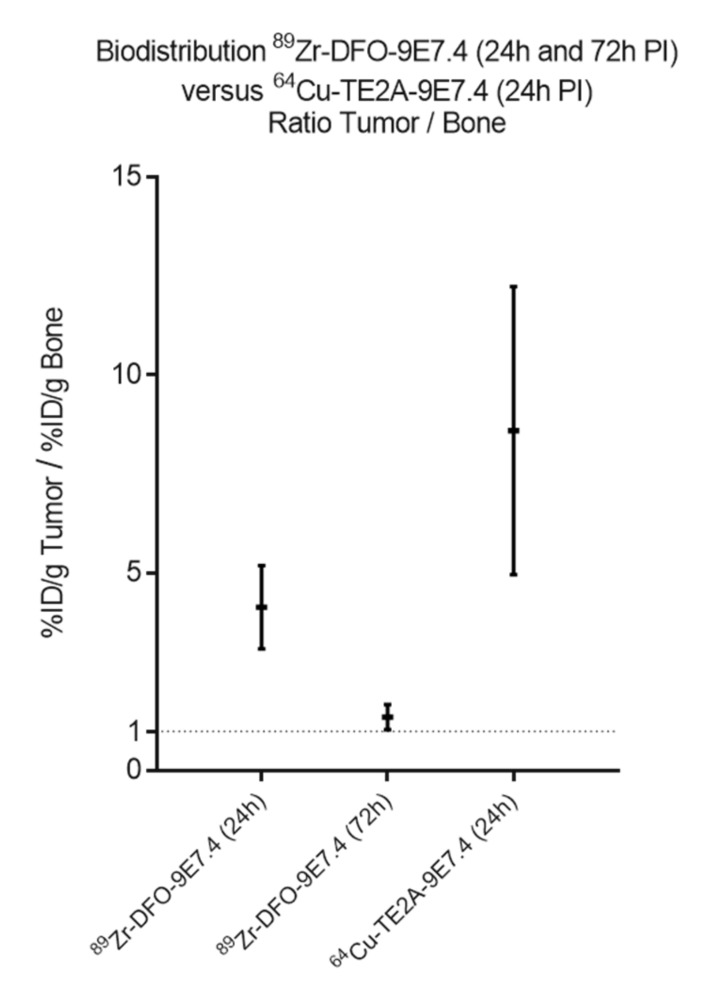
Tumor-to-bone ratios of ^89^Zr-DFO-9E7.4 and ^64^Cu-TE2A-9E7.4. Tumor-to-bone ratios of ^64^Cu-TE2A-9E7.4 at 24 h post-injection (PI) and ^89^Zr-DFO-9E7.4 at 24 h and 72 h PI in the subcutaneous tumor model (*n* = 3 for each group). Values are expressed in percentage of the injected radioactive dose per gram of tissue (%ID/g) and presented as mean ± SD.

Besides, despite similar levels of tumor and organ uptakes, few differences in the metabolization of the ^89^Zr- and ^64^Cu-labeled mAbs were observed at 24 h PI, as displayed in biodistribution experiments (Figure 8), extracted from Figure 1 and our recent published work [11]. ^89^Zr-DFO-9E7.4 showed similar uptakes than ^64^Cu-labeled 9E7.4 in the liver (13.92 ± 1.36 %ID/g versus 9.04 ± 0.36 %ID/g, respectively, at 24 h PI; *p* = 0.133; non-parametric test) and highest accumulation in bones (3.1 ± 1.15 versus 1.48 ± 0.29, respectively, at 24 h PI; *p* = 0.006; non-parametric test), spleen and blood. This notably resulted in net higher tumor to blood ratios for the ^64^Cu-immunoconjugate (4.08 ± 1.09 versus 1.42 ± 0.24, respectively, for ^64^Cu-TE2A-9E7.4 and ^89^Zr-DFO-9E7.4 at 24 h PI; *p* = 0.0391; non-parametric test). Similarly, significantly higher tumor to bone ratios for the ^64^Cu-labeled 9E7.4 were observed (Figure 9) (8.59 ± 3.64 for ^64^Cu-TE2A-9E7.4 at 24 h PI versus 4.13 ± 1.06 and 1.35 ± 0.32 for ^89^Zr-DFO-9E7.4 at 24 h and 72 h PI, respectively; *p* = 0.0127; non-parametric test).

## 3. Discussion

In recent years, immuno-PET established itself as a promising tool for personalized medicine in the context of multimodality treatment strategies [7]. In this context, the favorable properties of ^89^Zr for mAbs imaging have resulted in the growing interest and use of this isotope [17]. Given our past experiences with the anti-CD138 mAb 9E7.4 labeled with ^64^Cu [11], we evaluated in this present work ^89^Zr as an alternative radiolabel for proper imaging of MM tumors with 9E7.4. This study showed that ^89^Zr-DFO-9E7.4 binds effectively to CD138 tumors and allows MM imaging in a syngeneic mouse model (Figure 2, Figure 3 and Figure 4 and Figure 7). The radiotracer displayed good targeting properties, enabling high-contrast imaging as early as 24 h PI. The images showed excellent tumor to background ratios and although the contrast decreased at 48 h PI and 72 h PI, the tumors were clearly visible with ^89^Zr-DFO-9E7.4. The biodistribution data agreed well with the small animal PET results and showed ^89^Zr-DFO-9E7.4 peak uptake in the MM tumors at 24 h PI (12.47 ± 4.77 %ID/g) which decreased over time. Overall, ^89^Zr-DFO-9E7.4 presented markedly lower accumulation in non-target tissues with decreasing activity at 72 h PI, except for the liver, which displayed the second highest uptake at 24 h PI (13.92 ± 1.36 %ID/g) and showed longer tracer residency times (Figure 1). Only flat bones (2.97 ± 1.07 %ID/g at 24 h PI and 3.43 ± 1.12 %ID/g at 72 h PI) and femurs (3.22 ± 1.38 %ID/g at 24 h PI and 3.30 ± 0.70 %ID/g at 72 h PI) showed increasing activity between 24 h and 72 h PI (Figure 1). Such observations were not noted with the same antibody radiolabeled with 64Cu (data not published, observed between 24 h and 48 h PI) and were consistent with some preclinical studies describing the known in vivo gradual transchelation of ^89^Zr over time [18].

Indeed, to date, DFO is the most common chelator used in ^89^Zr-labeled radioimmuno-conjugates’ studies [17,20]. However, despite good stability of the ^89^Zr-DFO complex over a short period of time in preclinical studies, prolonged in vivo circulation times result in substantial release of ^89^Zr from DFO and increase of ^89^Zr uptake in the bones [18,21,22,23,24]. As seen by PET images, ^89^Zr bone depositions were markedly observed at the epiphyses of humerus, tibia and femur bones at 48 h and 72 h PI (Figure 3 and Figure 4), however also as early as 24 h PI. Due to a strong affinity for phosphate, ^89^Zr is steadily incorporated to hydroxyapatite, phosphates constituents of bones and particularly in the epiphyses where more active bone formation takes place [18,25]. This is illustrated by the bone dissection, PET and autoradiography imaging (Figure 4) where clear visual separation of the marrow cellular compartment with intra-medullary lesions from the mineralized bone seemed less evident at 72 h PI than 24 h PI. PET images with ^89^Zr-DFO-9E7.4 slowly yet surely “turned into” PET images with ^89^Zr-oxalate over time (Figure 3, Figure 4 and Figure 5). Yet, stability of the isotopes after chelation with conjugates and fate of free radionuclides are fundamental questions for isotope selection in the design of a radiotracer. Indeed, dissociation of the ^89^Zr-DFO complex could reduce the efficacy of an immuno-PET probe as an effective tool for bone lesions imaging as non-specific binding of free ^89^Zr at sites of osseous turnover could induce false-positive osseous foci. This potential drawback is illustrated in Figure 6 as multiple epiphyses false-positive osseous foci are observed in Mouse 17.

Thus, although ^89^Zr may hold great potential, this isotope is currently limited for both pre-clinical studies and clinical transition. In the oncological mouse model, many metastases occur in the metaphyses and epiphyses of the long bones. These sites conjugate active bone remodeling and high blood flow with fenestrated sinusoids which may predispose to tumor cells embolization and tumors growth in rodents [26,27]. Yet, with the “bone-seeking” nature of ^89^Zr making these localizations preferential sites for bone accumulation too, this could directly impact immuno-PET imaging and its use as a therapeutic planning companion. Indeed, multiple preclinical studies reported the use of ^89^Zr-labeled mAbs with elevated uptakes in the bones which (although not always described) clearly altered PET images’ interpretations [22,23,24,28,29]. These results are also in agreement with precedent findings of the use of immuno-PET as a scouting procedure before radioimmunotherapy (RIT) with large disparity of the distribution of ^89^Zr-conjugates and RIT conjugates [30,31,32]. Not to mention the fact that these preclinical studies were realized in human tumor xenografts models which usually overestimate tumor-uptake as being the sole source of antigen expression as opposed to the syngeneic mouse model.

In regards to clinical translation, ^89^Zr-labeled conjugates have shown minimal uptakes in bones in most of the few human studies found in the literature. Significantly slower bone turnover is indeed observed in comparison to rodents and ^89^Zr release is not expected to hinder the use of this radionuclide in patients [33,34,35,36]. Yet, recent works reporting a high level of false-positive suspicious ^89^Zr-trastuzumab–avid foci in patients with breast cancer [37,38] again caused concern for the translation of this radionuclide and chelator couple.

In this study, we have also opposed ^64^Cu-TE2A-9E7.4 and ^89^Zr-DFO-9E7.4 with the purpose of choosing the best tracer. As seen previously, free ^89^Zr and ^64^Cu from unstable chelates are known to accumulate in the bone and liver, respectively. Yet, our data showed similar uptakes of ^89^Zr-labeled and ^64^Cu-labeled 9E7.4 in the liver (13.92 ± 1.36 %ID/g versus 9.04 ± 0.36 %ID/g, respectively, at 24 h PI). At the same time point, bone uptakes of ^89^Zr-DFO-9E7.4 were higher than ^64^Cu-TE2A-9E7.4 (3.1 ± 1.15 versus 1.48 ± 0.29, respectively, at 24 h PI; *p* = 0.0061; non-parametric test) while the opposite was observed for tumor-to-blood ratio (1.42 ± 0.24 versus 4.08 ± 1.09, respectively, at 24 h PI; *p* = 0.0391; non-parametric test). Weighing these factors alone, ^64^Cu-labeled 9E7.4 appears as the best tracer for immuno-PET imaging. This conclusion is in agreement with the only other study which directly compared these two radionuclides in a preclinical model [39], however also with two recent works depicting the development of two immuno-PET tracers using CD38-directed human antibody daratumumab and ^89^Zr [40] and ^64^Cu [41], respectively, in xenograft MM mice model. Even if different methodologies were applied, the ^64^Cu-labeled radioconjugate seems to be the better choice. The development of better chelator agent for ^89^Zr as described in a recent review by Heskamp et al. [42] might change the face of the ranking in the future.

Consistent preclinical and clinical studies have been performed showing the potential for proper estimation of the probe biodistribution of immuno-PET before RIT [7]. Indeed, by translating tumor-to-background ratios into potential absorbed radiation doses, this approach allows for improved optimal dosing for personalized medicine in the context of multimodality treatment strategies. Moreover, ^64^Cu with the beta-emitting ^67^Cu provides an interesting theranostic pair with easy switch between diagnostic and therapeutic applications [16,43]. Up until now, due to its difficult production process, research on ^67^Cu is still restricted [16]. Nonetheless, the feasibility of anti-CD138 RIT was reported by our team with encouraging dosimetry results. CD138 targeting with a mAb coupled to a radionuclide emitting alpha particles also represents a potential new therapeutic option for MM and the use of alpha emitters with longer half-lives, such as 211At (7.2 h), should be evaluated in the clinic.

## 4. Materials and Methods

### 4.1. Cell Lines and Cultures

The 5T33 murine MM cell line that was used in this study was kindly provided by Dr. Harvey Turner (Nuclear Medicine Service, Fremantle Hospital, Western Australia) with the permission of Dr. Jiri Radl (TNO Institute, Leiden, Netherlands) [44]. Cells were transfected with luciferase cDNA as previously described [45]. Then, 5T33-Luc(+) were cultured in RPMI1640 medium (Gibco, Saint Aubin, France) containing 2 mM L-glutamine and 10% heat-inactivated fetal calf serum (PAA Laboratories / GE Healthcare Europe GmbH) at 37 °C, 5% CO_2_, 95% humidity.

### 4.2. Preparation of Immunoconjugates and Immuno-PET Tracers

#### 4.2.1. 9E7.4 Antibody

The 9E7.4 mAb was obtained by immunization of a rat with a 40-amino-acid peptide (GeneCust, Luxembourg, Luxembourg) derived from the murine CD138 protein (aa 90–130) (GenBank: CAA80254.1). Its characterization was ensured within the team as previously described [19]. The isotype of this antibody is IgG2a,κ, and its binding specificity is around 1 × 10^−10^ M.

#### 4.2.2. Labeling and Controls with ^89^Zr

DFO is the most common chelator used in ^89^Zr-labeled radioimmunoconjugates studies. P-isothiocyanatobenzyl-desferrioxamine B (DFO-SCN) was purchased from Macrocyclics (Dallas, USA). DFO-9E7.4 was prepared according to published protocols [20] by conjugating DFO-SCN to 9E7.4 mAb to secondly chelate ^89^Zr (t_1/2_ = 78.4 h; β^+^, 22.7%, E_β +_ max = 897 keV). Briefly, an aliquot of 4 mg/mL of 9E7.4 was prepared in borate buffered saline solution (0.3 M, pH 9.0). Freshly dissolved DFO-SCN in DMSO was added to the mixture in a 1:5 mAb:chelator molar ratio. The conjugation proceeded for 3 h at room temperature on an agitating block. The DFO-9E7.4 was purified from excess DFO-SCN by 50 kDa membrane dialysis using Amicon^®^ Ultra-15 (Merck Millipore, Darmstadt, Germany) with 0.25 M sodium acetate buffer (pH 5.5) as dialysis buffer and concentrated to 1.5 mg/mL.

For ^89^Zr labeling, under constant shaking, 185 MBq of ^89^Zr-oxalate was adjusted to pH 7.2 in HEPES buffer (0.5 M) and Na_2_CO_3_ (2 M). Finally, 330 µL of DFO-9E7.4 (1.5 mg/mL) was added and the pH was readjusted to 7.5 using HEPES buffer (0.5 M). The mixture was incubated at room temperature for 60 min. Radiochemical purity was determined by thin layer chromatography ITLC-SG using a citrate buffer (pH 4.5; 0.1 M) and was 93.8%. The ^89^Zr-labeled immuno-conjugate was thus secondly purified by size exclusion chromatography using a PD-10 column (Sephadex G25, GE Healthcare, Chicago, IL, United States). The radiochemical purity was finally assessed by ITLC-SG. The immunoreactivity of ^89^Zr-DFO-9E7.4 was determined using magnetic beads (Pierce, Thermo Scientific, Waltham, MA, United States) labeled with a 40 amino acids peptide recognized by the 9E7.4 antibody according to the supplier’s protocol. The radiochemical purity and its immunoreactivity were 98% and 80 ± 5%, respectively. At the end of the radiolabeling, the specific activity for ^89^Zr-DFO-9E7.4 was 5 ± 0.8 MBq/100µL (116 ± 13 MBq/mg).

#### 4.2.3. Labeling and Controls with ^64^Cu

Copper-64 (t_1/2_ = 12.7 h; β^+^, 17.8%, E_β +_ max = 656 keV; β^-^, 38.4%, E_β −_ max = 573 keV) was obtained from the ARRONAX cyclotron (GIP ARRONAX, Saint-Herblain, France) using the reaction ^64^Ni(p,n)^64^Cu and was delivered as ^64^CuCl_2_ in HCl 0.1N. Preparation and radiolabeling of ^64^Cu-TE2A-9E7.4 was carried out following our standard procedure and as previously reported in a recent work [11]. The radiolabeling yield and specific activity post-purification of the bioconjugate were 95 ± 2.8% and 188 ± 27 MBq/mg, respectively, and its immunoreactivity was 81 ± 7%.

#### 4.2.4. Preparation of ^89^Zr Oxalate

The supplied ^89^Zr-oxalate was neutralized with Na_2_CO_3_ (2 M) and diluted with saline (0.9% NaCl) to give a final oxalate concentration of 10 mM.

### 4.3. Animal Studies

#### 4.3.1. Animal Model: Subcutaneous Tumor Model and IV Disseminated Tumor Model

Female C57BL/KalwRij mice were purchased from Envigo and housed under conventional conditions at the Experimental Therapeutic Unit animal facility (SFR François Bonamy, IRS‑UN, University of Nantes, license number: B-44-278). Experiments were approved by the local veterinary committee (reference 00143.01, 28 march 2014) and carried out in accordance with relevant guidelines and regulations. Mice were 17 weeks old at the time of the experiments.

Twelve mice were grafted subcutaneously (SC) with 2.10^6^ 5T33-Luc(+) cells suspended in 100 μL of PBS 20 days before the first PET images. Six mice were grafted on the right leg and six on both legs. Tumors were grown to a size of 0.3–0.8 cm in diameter in line with ethical consideration in animal experiments.

To establish the optimal radiolabeled 9E7.4 mAb for bone lesions immuno-PET in MM, a well-known experimental disseminated model was generated: 1.10^6^ 5T33-Luc(+) cells were suspended in 100 μL of PBS and injected via the tail vein into seven mice 34 days before the first PET images. Mice were monitored for bone marrow lesions by bioluminescence imaging over 33 days.

#### 4.3.2. Bioluminescence Imaging

Mice were serially imaged using 2D bioluminescence imaging as previously described [14] to locate tumor progression.

The mice were anesthetized with intraperitoneal injection of 100 µL/10g of an anesthetic solution (consisting of 1 mL ketamine at 100 mg/mL (Panpharma); 0.5 mL xylazine at 20 mg/mL (Bayer); and 8.5 mL PBS). Mice were injected intraperitoneally with 100µL of luciferin (Interchim, 12 mg/mL) 5 min before being imaged. Mice were imaged in ventral and dorsal positions using a Photon IMAGER ™ (Biospace Lab, Paris, France) for 30 s. The images were analyzed using the M3Vision ™ software (Biospace Lab, Paris, France).

#### 4.3.3. Small Animal ImmunoPET-CT Imaging

PET-CT preclinical exams were performed on our local imaging center, CIMA (Centre d’Imagerie Multimodale Appliquée, Nantes, France).

For the SC model, nine mice were imaged with ^18^F-FDG-PET and ^89^Zr-DFO-9E7.4 (Mice 1 to 9) and three with ^18^F-FDG-PET and ^89^Zr-Oxalate (Mice 10 to 12).

For the disseminated model, five mice were imaged with ^18^F-FDG-PET and ^89^Zr-DFO-9E7.4 (Mice 13 to 17). Mice 18 and 19 were imaged with ^18^F-FDG-PET and ^64^Cu-TE2A-9E7.4 in a first session and secondly imaged one week later with ^18^F-FDG-PET and ^89^Zr-DFO-9E7.4.

One mouse (Mouse 20) with no graft was imaged with ^89^Zr-Oxalate as control imaging for this radionuclide.

For ^18^F-FDG-PET imaging, mice were fasted overnight (6 h to 12 h) with free access to water. Mice were warmed for at least one hour, anesthetized with inhaled isoflurane 2.5% and intravenously injected with 10 MBq of ^18^F-FDG in a volume of 100 μL through the lateral tail vein. Mice were maintained under anesthesia for a 1 h uptake period and then scanned (350–650 keV energy window, 20 min listmode acquisition, 3D rebinning followed by OSEM-MAP reconstruction) on a multi-modality preclinical imaging system (Inveon™, Siemens Healthcare, Erlangen, Germany). CT acquisitions (80 kV, 0.5 mA) were also performed immediately before the PET imaging. The reconstructed PET images were analyzed using Inveon Research Workplace (Siemens Healthcare).

For ^89^Zr PET studies, similar procedures were followed 24 h post-^18^F-FDG-PET imaging, except that no fasting was performed and imaging occurred at 24 h, 48 h and 72 h post-injection (PI). Considering the long physical half-life of ^89^Zr and the pharmacokinetic profile of antibody-based radiotracers [11], time points before 24 h were not realized for longitudinal PET imaging studies. Each mouse was intravenously injected with 5 MBq of radiotracer (^89^Zr-DFO-9E7.4 or ^89^Zr-Oxalate) in a volume of 100 μL via the lateral tail vein. According to the ^89^Zr decay, the specific activity at the injection time was between 116 MBq/mg and 180 MBq/mg for ^89^Zr-DFO-9E7.4.

For ^64^Cu PET studies, similar procedures were followed and imaging occurred at 24 h PI. Mice were intravenously injected with 10 MBq of ^64^Cu-TE2A-9E7.4 in a volume of 100 μL via the lateral tail vein. According to the ^64^Cu decay, the specific activity at the injection time was between 140 MBq/mg and 170 MBq/mg.

#### 4.3.4. Biodistribution Study

Tracer biodistribution studies were carried out in all the SC-tumor-bearing mice after PET imaging (*n* = 3 for each group): at 24 h and 72 h PI for ^89^Zr-DFO-9E7.4 and at 24 h PI for ^89^Zr-oxalate. Tumor, blood and other selected tissues (liver, kidney, gut, lungs, muscle, spleen, skin, brain, heart, flat bone, femur and stomach) were dissected, weighed and counted on a calibrated and normalized gamma-counter. For each organ, the percentage of injected dose per gram (%ID/g) was calculated. The organ to blood ratios were also compared.

#### 4.3.5. Digital Autoradiography

Femurs of Mice 14, 15 and 20 were removed, fast-frozen in cold 2-methylbutane solution, embedded in optimal-cutting temperature compound and cut into 10 µm sections using a cryomicrotome (CM3050 Leica Biosystems®). Sections were mounted on Superfrost™ slides and digital autoradiography images were obtained on a BeaQuant - Real-time autoradiography (Ai4R, Nantes, France). Image analysis was performed on the dedicated software Beamage^®^ (version 1.0, Ai4R, Nantes, France).

### 4.4. Statistical Analysis

Statistical analysis was performed using GraphPad Prism version 7.00. Differences in uptake were tested for significance using the non-parametric Mann-Whitney test for two groups. A *p* value below 0.05 was considered significant.

## 5. Conclusions

In this study, we synthetized ^64^Cu- and ^89^Zr-labeled anti-CD138 antibodies that were able to detect subcutaneous MM tumors and bone marrow lesions with high sensitivity, outperforming ^18^F-FDG-PET in this preclinical model. In a theranostic approach, the stability issue and “bone-seeking” nature of ^89^Zr could directly impact immuno-PET imaging and its use as a therapeutic planning companion. Imaging ^64^Cu-anti-CD138 antibody indeed had the most optimal tumor-to-non-target tissue ratios for translation into humans as a specific and promising new imaging radiopharmaceutical agent in MM. These data also support ^64^Cu-TE2A-9E7.4 as a promising imaging tool for selecting patients before the realization of RIT.

## Figures and Tables

**Table 1:** Biodistribution results and organ-to-blood ratios of ^89^Zr-DFO-9E7.4, ^89^Zr-oxalate in tumor-bearing mice. Ex vivo biodistribution results and organ-to-blood ratios of ^89^Zr-DFO-9E7.4 at 24 h and 72 h post-injection (PI), in the subcutaneous tumor model (*n* = 3 for each group). Ex vivo biodistribution results and organ-to-blood ratios of ^89^Zr-oxalate at 24 h PI (*n* = 3). Values are expressed in percentage of the injected radioactive dose per gram of tissue (%ID/g) and presented as mean +/- SD.

Organs	^89^Zr-DFO-9E7.4 (24 h PI)		^89^Zr-DFO-9E7.4 (24 h PI)		^89^Zr-DFO-9E7.4 (72 h PI)		^89^Zr-DFO-9E7.4 (72 h PI)		^89^Zr-Oxalate (24 h PI)		^89^Zr-Oxalate (24 h PI)	
			Ratio Organs/Blood				Ratio Organs/Blood				Ratio Organs/Blood	
	Median	SD	Median	SD	Median	SD	Median	SD	Median	SD	Median	SD
Tumor	12.48	4.77	1.42	0.47	4.53	1.76	3.42	0.22	9.67	2.52	0.83	0.15
Blood	5.33	0.95	1.00	0.00	1.77	0.85	1.00	0.00	11.57	1.38	1.00	0.00
Liver	13.93	1.36	2.30	1.00	9.90	1.75	6.44	2.55	8.60	0.53	0.75	0.13
Kidney	5.78	2.83	0.62	0.13	3.60	1.37	2.15	0.61	12.33	1.53	1.09	0.27
Gut	7.03	0.95	1.34	0.41	6.13	2.47	3.65	0.98	5.47	1.29	0.47	0.06
Lungs	5.70	2.79	0.86	0.22	2.77	1.14	1.60	0.16	7.90	0.79	0.69	0.01
Muscle	1.05	0.49	0.11	0.03	0.27	0.15	0.16	0.04	2.63	0.21	0.23	0.01
Spleen	10.78	1.86	2.00	0.56	7.23	1.01	4.85	2.36	10.90	1.01	0.96	0.21
Skin	3.28	0.90	0.41	0.25	1.40	0.69	0.80	0.18	3.77	0.15	0.33	0.05
Brain	0.68	0.71	0.05	0.03	0.20	0.17	0.20	0.25	0.50	0.10	0.04	0.01
Heart	4.60	2.81	0.45	0.02	0.93	0.64	0.46	0.29	8.70	1.13	0.77	0.20
Flat Bone	2.98	1.07	0.34	0.12	3.43	1.12	2.11	0.60	39.20	2.03	3.44	0.58
Femur	3.23	1.38	0.45	0.11	3.30	0.70	2.16	0.63	38.43	1.40	3.37	0.55
Stomach	5.30	1.35	0.62	0.46	2.77	0.45	1.82	0.76	14.67	1.53	1.29	0.28

## Supplementary Materials

Supplementary materials can be found at https://www.mdpi.com/1422-0067/20/10/2564/s1.

## Abbreviations

^64^Cu	copper-64
DFO	desferrioxamine B
DFO-SCN	P-isothiocyanatobenzyl-desferrioxamine B;
^18^F-FDG	[18]-Fluorodeoxyglucose
mAb	monoclonal antibody
MM	multiple myeloma
PET	positron emission tomography
PI	post-injection
RIT	radioimmunotherapy
SC	subcutaneously
TE2A	TE2A-benzyl isothiocyanate
^89^Zr	Zirconium-89
