# Supplementary material for: What is the Best Radionuclide for Immuno-PET of Multiple Myeloma? A Comparison Study Between 89Zr- and 64Cu-Labeled Anti-CD138 in a Preclinical Syngeneic Model
Source: Int J Mol Sci. 2019 May 24;20(10):2564. doi: 10.3390/ijms20102564 (PMC6567828; doi:10.3390/ijms20102564)
Supplement: Supplementary file 1 [file ijms-20-02564-s001.pdf]

Supplemental Data:

Table S1. Different PET imaging conducted in mice and lesions visualized on the different imaging methods

	**Bioluminescence imaging**	**^{18}F-FDG-PET**	**^{89}Zr -DFO-9E7.4**	**^{89}Zr-oxalate**	**^{64}Cu-TE2A-9E7.4**
	**Subcutaneous tumor model**				
Mouse 1		Sc x 1	Sc x 1		
Mouse 2		Sc x 1,	Sc x 1,		
Mouse 3		Sc x 1	Sc x 1		
Mouse 4		Sc x 1	Sc x 1		
Mouse 5		Sc x 1, Ln	Sc x 1, Ln		
Mouse 6		Sc x 1	Sc x 1		
Mouse 7		Sc x 1	Sc x 1		
Mouse 8		Sc x 1,	Sc x 1,		
Mouse 9		Sc x 1	Sc x 1		
Mouse 10		Sc x 2		Sc x 2	
Mouse 11		Sc x 2		Sc x 2	
Mouse 12		Sc x 2		Sc x 2	
	**IV disseminated tumor model**				
Mouse 13	Sa, Mb	Sa, Mb	Sa, Mb		
Mouse 14	Sk, Mb	Mb	Sk, Mb		
Mouse 15	Mb	Mb	Mb		
Mouse 16	Sk, Mb	Sk, Mb	Sk, Mb		
Mouse 17	Sk, Sa, Iw, Mb	Sk, Sa, Iw, Mb	Sk, Sa, Iw, Mb		
Mouse 18	Sk, Sp, Iw	Sk	Sk, Sp, Iw		Sk, Sp, Iw
Mouse 19	Sk, Sa, Mb	Sk, Sa	Sk, Sa, Mb		Sk, Sa, Mb
	**Control mouse**				
Mouse 20				N.S.	

Lesional territories: Sc: subcutaneous; Ln: lymph node; Sk: skull; Sa: Sacrum, Iw: iliac wing; Mb: member; Sp: spine.
